# Emergence and Inter- and Intrahost Evolution of Pandrug-Resistant Klebsiella pneumoniae Coharboring *tmexCD1-toprJ1, bla*_NDM-1_, and *bla*_KPC-2_

**DOI:** 10.1128/spectrum.02786-22

**Published:** 2023-01-31

**Authors:** Chao Liu, Pengcheng Du, Ping Yang, Jiajia Zheng, Juan Yi, Ming Lu, Ning Shen

**Affiliations:** a Department of Infectious Disease, Peking University Third Hospital, Beijing, China; b Center of Infectious Disease, Peking University Third Hospital, Beijing, China; c Qitan Technology Ltd., Chengdu, China; d Department of Pulmonary and Critical Care Medicine, Peking University Third Hospital, Beijing, China; e Institute of Medical Technology, Peking University Health Science Center, Beijing, China; f Department of Laboratory Medicine, Peking University Third Hospital, Beijing, China; Houston Methodist Hospital

**Keywords:** *tmexCD1-toprJ1*, within-host evolution, heterogeneity, pandrug resistance, whole-genome sequencing

## Abstract

Klebsiella pneumoniae is capable of acquiring various exogenous genetic elements and subsequently conferring high antimicrobial resistance. Recently, a plasmid-mediated RND family multidrug efflux pump gene cluster, *tmexCD1-toprJ1*, was discovered in K. pneumoniae. In this study, we analyzed tigecycline-resistant K. pneumoniae isolates from patients from surveillance from 2017 to 2021. In addition to phenotype detection, including growth curves, plasmid transferability and stability, hypermucoviscosity, biofilm formation, and serum survival, by whole-genome sequencing, we analyzed the phylogenetic relationships of the isolates harboring *tmexCD1-toprJ1* and discovered the composition of plasmids carrying *tmexCD1-toprJ1*. In total, we discovered that 12 tigecycline-resistant isolates from 5 patients possessed *tmexCD1-toprJ1*, designated sequence type 22 (ST22) and ST3691. An ST11 isolate harbored a partial *tmexD1*, and a complete *toprJ1* (*tmexC1* was lost) was tigecycline sensitive. All the ST22 tigecycline-resistant isolates coharbored *tmexCD1-toprJ1*, *bla*_NDM-1_, and *bla*_KPC-2_. *tmexCD1-toprJ1* was encoded by a novel IncU plasmid in ST22 and an IncFIB/HI1B plasmid in ST3691, which presented differences in mobility and stability. Interestingly, isolates from the same patients presented heteroresistance to tigecycline, not only among isolates from different specimens but also those from the same sample, which might be attributed to the differential expression of *tmexCD1-toprJ1* due to the dynamic genetic heterogeneity caused by relocating *tmexCD1-toprJ1* close to the replication origin of plasmid. Here, we reported the emergence of K. pneumoniae isolates coharboring *tmexCD1-toprJ1*, *bla*_NDM-1_, and *bla*_KPC-2_. The results highlight the impact of *in vivo* genetic heterogeneity of *tmexCD1-toprJ1*-carrying elements on the *in vivo* variation of tigecycline resistance, which might have notable influences on antimicrobial treatment.

**IMPORTANCE** Pandrug-resistant (PDR) Klebsiella pneumoniae poses a great challenge to public health, and tigecycline is an essential choice for antimicrobial treatment. In this study, we reported the emergence of PDR K. pneumoniae coharboring *tmexCD1-toprJ1*, *bla*_NDM-1_, and *bla*_KPC-2_, which belongs to ST22 and ST3691. By whole-genome analysis, we reconstructed the evolutionary map of the ST22 ancestor to become the PDR superbug by acquiring multiple genetic elements encoding *tmexCD1-toprJ1* or *bla*_NDM-1_. Importantly, the genetic contexts of *tmexCD1-toprJ1* among the ST22 isolates are different and present with various mobilities and stabilities. Furthermore, we also discovered the heterogeneity of tigecycline resistance during long-term infection of ST22, which might be attributed to the differential expression of *tmexCD1-toprJ1* due to the dynamic genetic heterogeneity caused by relocating *tmexCD1-toprJ1* close to the replication origin of plasmid. This study tracks the inter- and intrahost microevolution of the superbug PDR K. pneumoniae and highlights the importance of timely monitoring of the variation of pathogens during antimicrobial treatment.

## INTRODUCTION

Klebsiella pneumoniae is becoming increasingly prevalent in China, posing a great threat to public health (1). Notably, K. pneumoniae is capable of acquiring various exogenous genetic elements and subsequently conferring multidrug resistance (MDR) or pandrug resistance (PDR) phenotypes, playing a key role in survival within various environments (2, 3). Antimicrobial resistance (AMR) transferred by mobile genetic elements is essential for adaptation to antibiotic selection pressure.

Tigecycline is regarded as the last-resort antimicrobial to treat serious infections caused by carbapenem-resistant (CR) bacteria (4). A previous study reported that chromosomal mutations and overexpression of efflux pumps were closely associated with tigecycline resistance (5). After the plasmid-borne *tet*(X) family genes were identified, tigecycline-resistant bacteria have been rapidly discovered within *Enterobacteriaceae* (6–8). Moreover, a plasmid-mediated RND family multidrug efflux pump gene cluster, *tmexCD1-toprJ1*, was also discovered in K. pneumoniae (9). At present, the *tmexCD1-toprJ1*-like-positive K. pneumoniae strains have been mostly discovered in China (9, 10) and Vietnam (11), widely isolated from patients, animals, and food, undermining the efficacy of tigecycline (10, 12–14). Additionally, the *tmexCD1-toprJ1-like* gene cluster also emerged within Raoultella ornithinolytica (15), Pseudomonas putida (13), and Klebsiella quasipneumoniae (16), which are common in health care settings. Moreover, mutants of the *tmexCD1-toprJ1* gene cluster and *tnfxB3-tmexCD3-toprJ1b* were subsequently reported in carbapenem-resistant and colistin-resistant strains (13, 15, 17–20).

Here, we conducted retrospective genomic surveillance in our hospital to investigate the prevalence and genomic characteristics of *tmexCD1-toprJ1*-positive K. pneumoniae isolates. We found that although the *tmexCD1-toprJ1*-positive K. pneumoniae isolates were rare, the *tmexCD1-toprJ1* gene clusters were located in two types of plasmids harbored by K. pneumoniae strains of two different sequence types (STs). Importantly, ST22 K. pneumoniae, coharboring *tmexCD1-toprJ1*, *bla*_NDM-1_, and *bla*_KPC-2_, emerged in the hospital and caused a fatal infection. Furthermore, the plasmid harboring *tmexCD1-toprJ1* displayed heterogeneity not only among isolates from continually collected specimens from the same host but also among those from the same specimen, which might be associated with the differential expression of *tmexCD1-toprJ1* and selective antibiotic pressure.

## RESULTS

### Clinical and microbiological characteristics of tigecycline-resistant K. pneumoniae.

**FIG 1:**
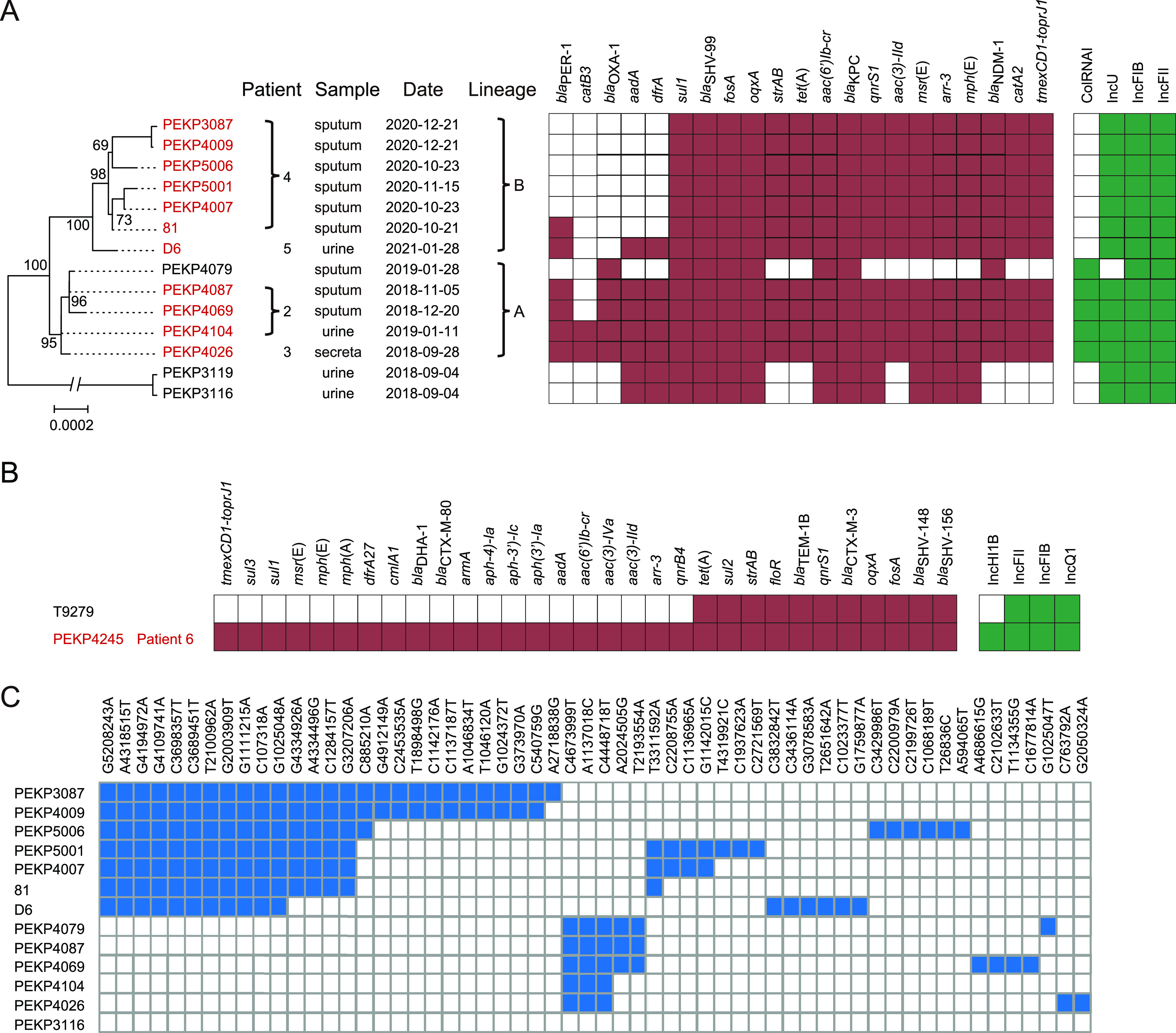
Phylogenetic relationship and molecular characteristics of tigecycline-resistant K. pneumoniae strains in this study. (A) Phylogenetic tree (left), carriage of resistance genes (middle), and types of plasmid replicons (right) of our ST22 K. pneumoniae and published ST22 genomes. The colored block represents the presence of each element. (B) Carriage of resistance genes (left) and types of plasmid replicons (right) of our ST3691 K. pneumoniae and published ST22 genomes. The colored block represents the presence of each element. (C) SNPs among tigecycline-resistant ST22 using PEKP3116 as a reference. The mutation positions and bases (reference base, position, mutant) are marked on the top of the heatmap. The colored blocks represent the existence of mutations.

During the surveillance, we obtained 12 tigecycline-resistant K. pneumoniae isolates from 5 patients among 720 available K. pneumoniae isolates from 454 patients in total. Two of the five patients were diagnosed as having community-acquired infection and the others as having hospital-acquired infection (Table 1). Three isolates (PEKP4087, PEKP4069, and PEKP4104) were isolated from patient 2, and six isolates (81, PEKP4007, PEKP5006, PEKP5001, PEKP3087, and PEKP4009) were isolated from sputum samples continually collected from patient 4. Isolates PEKP4007 and PEKP5006 were from two different sputum samples collected on the same day, which is the same condition as for PEKP3087 and PEKP4009 (Fig. 1). The MICs of tigecycline against these isolates ranged from 4 to 24 mg/L. Eleven isolates exhibited a PDR phenotype, and one (PEKP4245) showed MDR to most of the antibiotics, including ceftazidime-avibactam, aminoglycosides, quinolones, β-lactamase inhibitor, and carbapenems (see Table S1 in the supplemental material). Two patients infected by PDR isolates died within 30 days after the last isolation (Table 1 and Fig. 1A and B).

**TABLE 1:** Characteristics of the *tmexCD1-toprJ1*-like gene cluster strains^*f*^

Characteristic	Data for patient:					
	1^*a*^	2	3	4	5	6
Age (yrs)	36	64	81	83	30	52
Sex	Female	Male	Male	Female	Female	Male
Specimen	Tissue	^*b*^	Wound	Sputum	Urine	Urine
Isolation date (yr-mo-day)	2020-11-09	^*c*^	2018-09-28	^*c*^	2021-01-28	2019-07-07
Underlying disease(s)	Diabetes, COPD, cerebrovascular disease	Hypertension, COPD, urinary diseases	CKD, rhabdomyolysis	Heart failure, digestive diseases	Digestive diseases, hypothyroidism	Poliomyelitis
Infection type	CAI	HAI	HAI	HAI	HAI	CAI
Antibiotic agent exposure	P	P	P	P	P	P
Intubation	N	Stomach tube, urinary tube, CVC, tracheal intubation	Stomach tube, CVC, urinary tube	Stomach tube, urinary tube	Stomach tube, urinary tube	Urinary tube
CCI	4	3	2	2	3	1
Life-sustainable strategy	N	Mechanical ventilation	N	Mechanical ventilation	N	N
Severity	N	Sepsis	N	N	Septic Shock	N
Outcome	Survived	Death	Survived	Death	Survived	Survived
Strain(s)	PEKP3038	PEKP4087, PEKP4069, PEKP4104	PEKP4026	81, PEKP4007, PEKP5006, PEKP5001, PEKP3087, PEKP4009	D6	PEKP4245
ST	ST11	ST22	ST22	ST22	ST22	ST3691
Key AMR	*bla*_DHA-1_, *bla*_CTX-M-15_	*bla*_KPC-2_, *bla*_NDM-1_, *bla*_OXA-1_	*bla*_KPC-2_, *bla*_NDM-1_, *bla*_OXA-1_	*bla*_KPC-2_, *bla*_NDM-1_, *MgrB*^*d*^	*bla*_KPC-2_, *bla*_NDM-1_, *MgrB*^*d*^	*bla*_DHA-1_
Biofilm	Weakly positive	Weakly positive	Weakly positive	Weakly positive	Positive	Weakly positive
Serum killing assay	Sensitive	Sensitive	Sensitive	Sensitive	Sensitive	Sensitive
Hypermucoviscosity	N	P^*e*^	N	N	N	N
Serotype	KL10	KL9	KL9	KL9	KL9	KL46
O_locus	Unknow	O2v2	O2v2	O2v2	O2v2	O3b

a Strains harboring *tmexD1-toprJ1*.

b PEKP4087 and PEKP4069 were from sputum, and PEKP4104 was from urine.

c Two patients presented with continuous ST22 strain isolation (Fig. 1).

d *MgrB* was truncated.

e PEKP4069 presented with hypermucoviscosity.

f COPD, chronic obstructive pulmonary disease; CKD, chronic kidney disease; CAI, community-acquired infection; HAI, hospital-acquired infection; CVC, central venous catheter; N, negative; P, positive.

### Molecular characteristics, features of plasmid types, and resistance genes.

Further whole-genome sequencing (WGS) and analysis revealed the molecular characteristics of the isolates that correspond to the observed phenotypes. The 11 PDR isolates among the 12 tigecycline-resistant K. pneumoniae belonged to ST22, and the one MDR isolate was ST3691. All of the isolates harbored *tmexCD1-toprJ1*. A tigecycline-sensitive ST11 isolate (PEKP3038) harbored truncated *tmexD1* and *toprJ1*, and *tmexC1* was lost. We also obtained the genomes of three tigecycline-sensitive ST22 isolates (PEKP4079, PEKP3116, and PEKP3119) and an ST3691 isolate (PEKP4245), which were *tmexCD1-toprJ1* negative. All 14 ST22 isolates harbored IncFIB- and IncFII-type plasmid replicons (Fig. 1A). Most of them (13/14) also harbored the IncU-type plasmid replicon, including two isolates without *tmexCD1-toprJ1*. Compared with the *tmexCD1-toprJ1*-negative isolates, all 11 tigecycline-resistant ST22 isolates harbored resistance genes, including *strAB*, *tet*(A), *aac(3)-IId*, *bla*_NDM-1_, and *catA2* in addition to *tmexCD1-toprJ1*. Similarly, compared with the *tmexCD1-toprJ1*-negative ST3691 isolate, the *tmexCD1-toprJ1*-carrying ST3691 isolate PEKP4245 comprised an extra IncH1B plasmid replicon and a number of resistance genes (Fig. 1B). The distribution of resistance genes was in line with the resistance phenotypes (Table S2).

### Genetic characteristics of *tmexCD1-toprJ1*-bearing plasmids.

**FIG 2:**
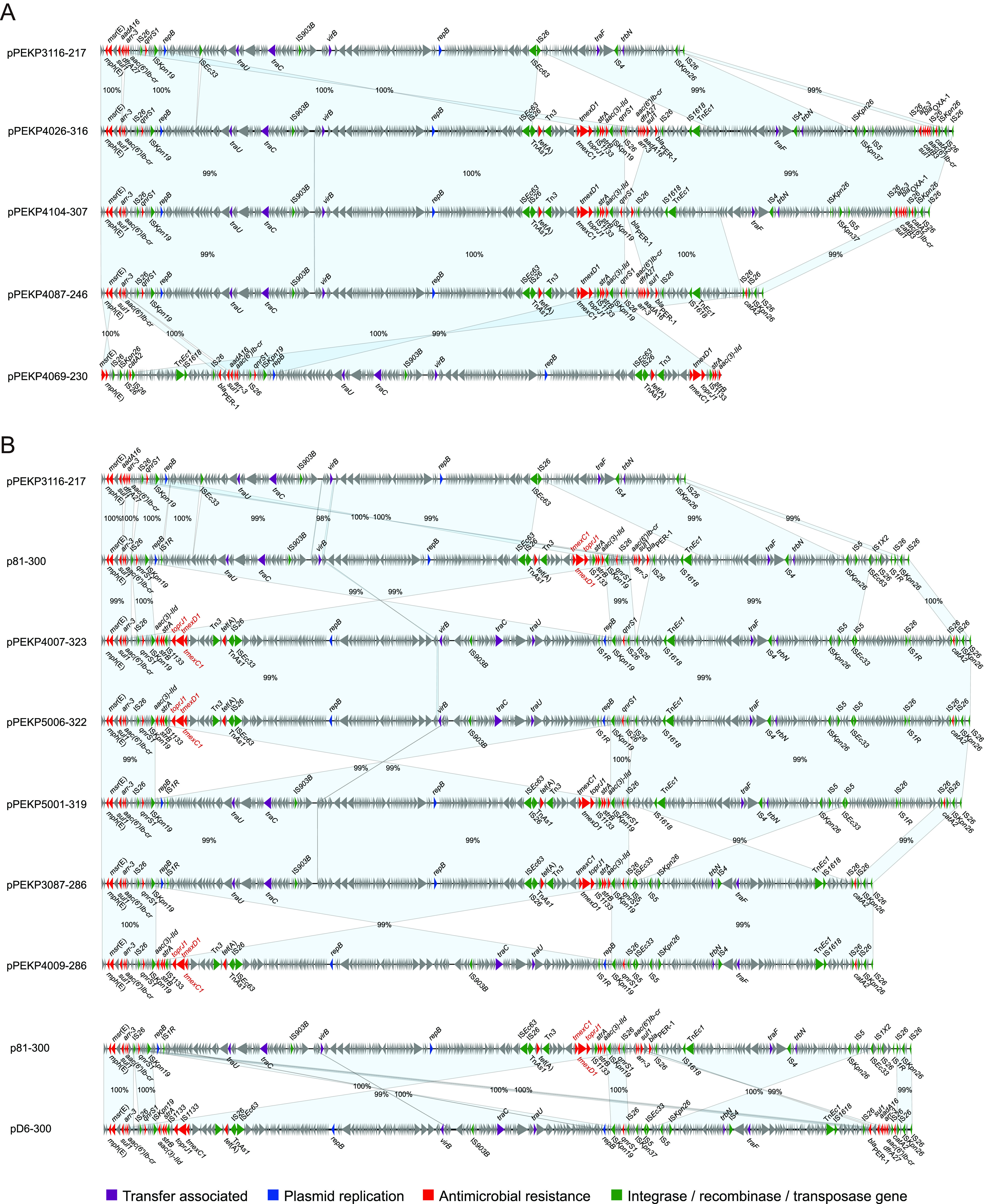
Sequence alignments of *tmexCD1-toprJ1*-bearing IncU plasmids of tigecycline-resistant ST22 K. pneumoniae within lineage A (A) and lineage B (B). The matched regions between two sequences are displayed by light blue blocks, and the identities are marked. The arrows represent the genes related to resistance and transfer (red, AMR; green, integrase recombinase and transposase; purple, transfer associated; dark blue, plasmid replication; gray, other functions).

**FIG 3:**
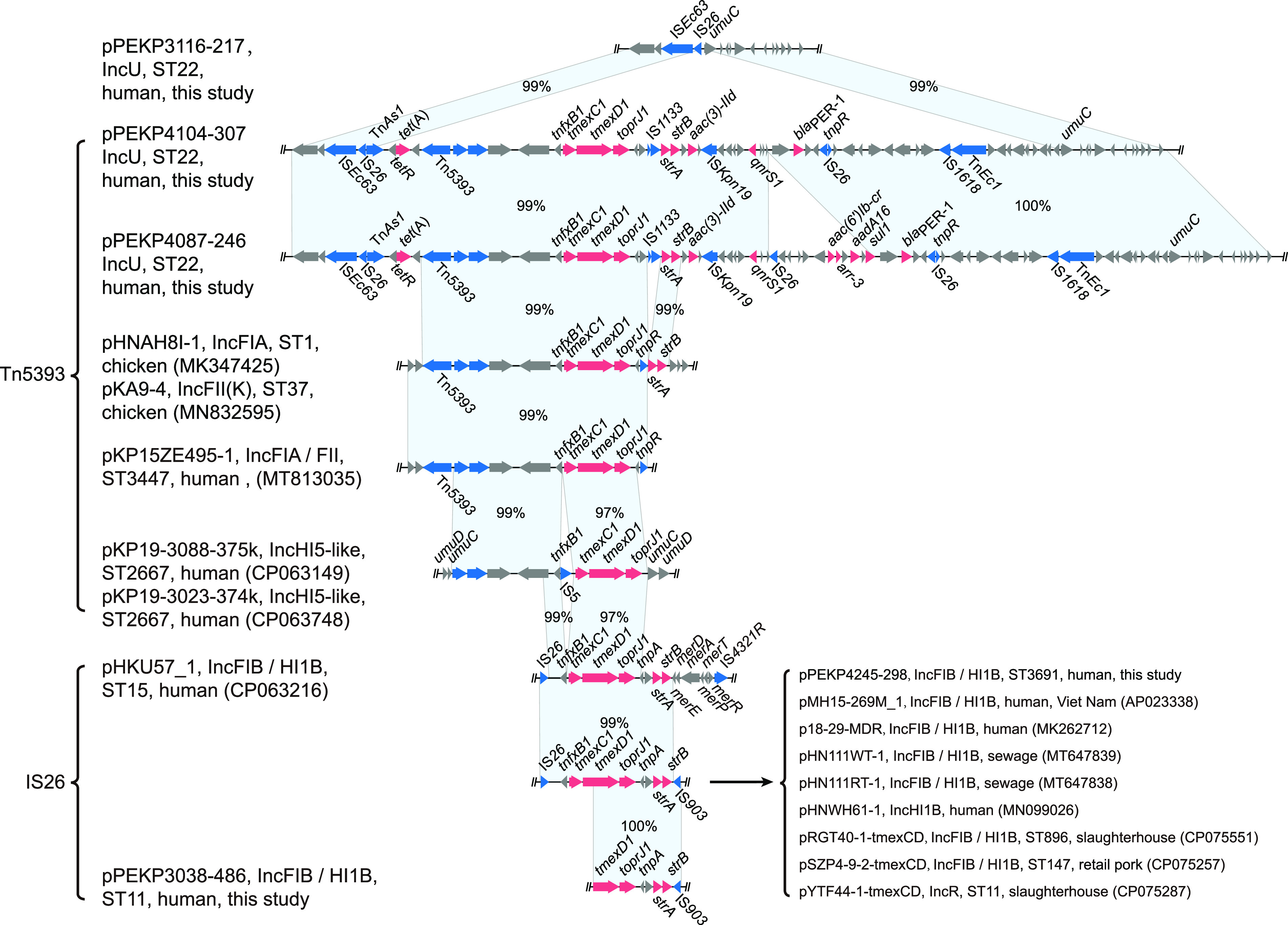
Genetic contexts and distribution of *tmexCD1-toprJ1*-bearing elements. (A) Genetic contexts and alignments of *tmexCD1-toprJ1*-bearing elements. The matched regions between two sequences are displayed by light blue blocks, and the identities are marked. The arrows represent the genes related to resistance and transfer (red, AMR; blue, integrase recombinase and transposase; gray, other functions). The plasmid names, types of plasmid replicons, sequence types of the K. pneumoniae strains, and sources of the strains are marked.

**FIG 4:**
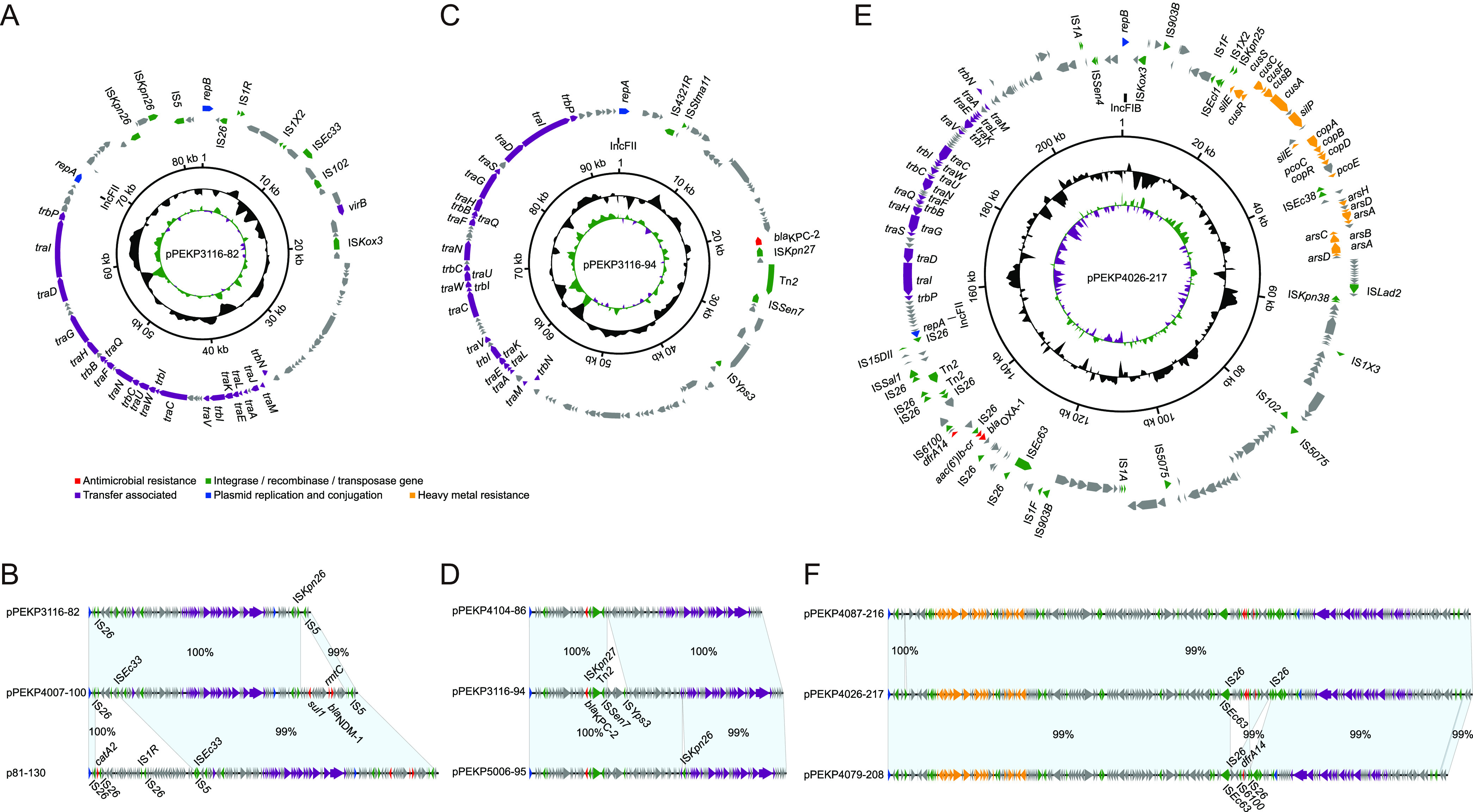
Circular sketch map and alignments of plasmids harboring *bla*_NDM-1_ (A and B), *bla*_KPC-2_ (C and D), and heavy metal resistance genes (E and F) from ST22 K. pneumoniae strains. The matched regions between two sequences are displayed by light blue blocks, and the identities are marked. The arrows represent the genes related to resistance and transfer (red, AMR; green, integrase recombinase and transposase; purple, transfer associated; dark blue, plasmid replication; orange, heavy metal resistance; gray, other functions).

To explore the characteristics of *tmexCD1-toprJ1*-bearing genetic elements, we obtained the complete circularized sequences of chromosomes and plasmids of these isolates. We found that the *tmexCD1-toprJ1* gene clusters were carried by two types of plasmids. The plasmids from ST22 isolates were highly similar (Fig. 2). These plasmids were ~230 to 323 kb in size and comprised the IncU replicon, similar to two *tmexCD1-toprJ1* negative plasmids (pPEKP3116-217 and pPEKP3119-216, with ~80.44 to 99.89% coverage and ~97.07 to 99.80% identity). We identified two major insertions in *tmexCD1-toprJ1*-bearing IncU plasmids. The first one was adjacent to an insertion sequence 26 (IS*26*) element encoding *tmexCD1-toprJ1* and six other resistance genes, and the other insertion encoding *arr-3* and *bla*_OXA-1_ was adjacent to an IS*Kpn26* element (Fig. 2). The core genetic environment of *tmexCD1-toprJ1* comprised *Tn501-int-int-hp-hp-tnfxB1-tmexC1-tmexD1-toprJ1* (Fig. 3A). This composition was most similar to the corresponding sequences of the IncFIA plasmid pHNAH8I-1 of ST1 K. pneumoniae and the IncFII(K) plasmid pKA9-4 of ST37 K. pneumoniae isolated from chickens in China (Fig. S1) (10, 13). In addition, this structure was located within the large insertion adjacent to IS*26* and comprised other resistance genes, transposase genes, and ISs, suggesting that this novel IncU-type *tmexCD1-toprJ1*-bearing plasmid might be generated via multiple steps of recombination. In addition, tigecycline-resistant ST22 also harbored multiple IncF plasmids encoding *bla*_KPC-2_, *bla*_NDM-1_, and heavy metal resistance genes (Fig. 4).

The *tmexCD1-toprJ1*-bearing plasmid pPEKP4245-298 from the ST3691 isolate and plasmid pPEKP3038-486 carrying truncated *tmexD1* and *toprJ1* from the ST11 isolate were similar to previously reported *tmexCD1-toprJ1*-bearing plasmids (Fig. S2). The core genetic environment comprised *IS26-tnfxB1-tmexC1-tmexD1-toprJ1-tnpA-strA-strB-IS903* in plasmid pPEKP4245-298 and similar published plasmids (Fig. 3A), and the *IS26*, *tmexC1*, and 5′ end of *tmexD1* were truncated in plasmid pPEKP3038-486.

### Inter- and intrahost genetic heterogeneity and microevolution of the ST22 population.

To explore the evolutionary history of the ST22 isolates from our hospital, we further analyzed the phylogenetic relationships and genetic differences. The *tmexCD1-toprJ1*-harboring isolates clustered together with three *tmexCD1-toprJ1*-negative isolates (Fig. S1D). One of the three *tmexCD1-toprJ1*-negative isolates, PEKP4079, showed only one nucleotide difference compared with the *tmexCD1-toprJ1*-harboring isolate PEKP4087. Between two other *tmexCD1-toprJ1*-negative isolates, PEKP3116 and PEKP3119, only two single nucleotide polymorphisms (SNPs) were identified, which could be regarded as the same clone. Meanwhile, we found 549 shared SNPs within the *tmexCD1-toprJ1*-harboring cluster compared with PEKP3116 and PEKP3119 (Table S1). In contrast, only 1 to 11 SNPs were found among the *tmexCD1-toprJ1*-harboring cluster. We also identified 2 lineages among the *tmexCD1-toprJ1*-harboring isolates, differing by 11 common SNPs to each other (Fig. 1A and C). Lineage A included the isolates from patient 3 (PEKP4026) and patient 2 (PEKP4104, PEKP4087, and PEKP4069), and lineage B included the isolates from patient 4 (81, PEKP4007, PEKP5001, PEKP5006, PEKP4009, and PEKP3087) and patient 5 (D6).

**FIG 5:**
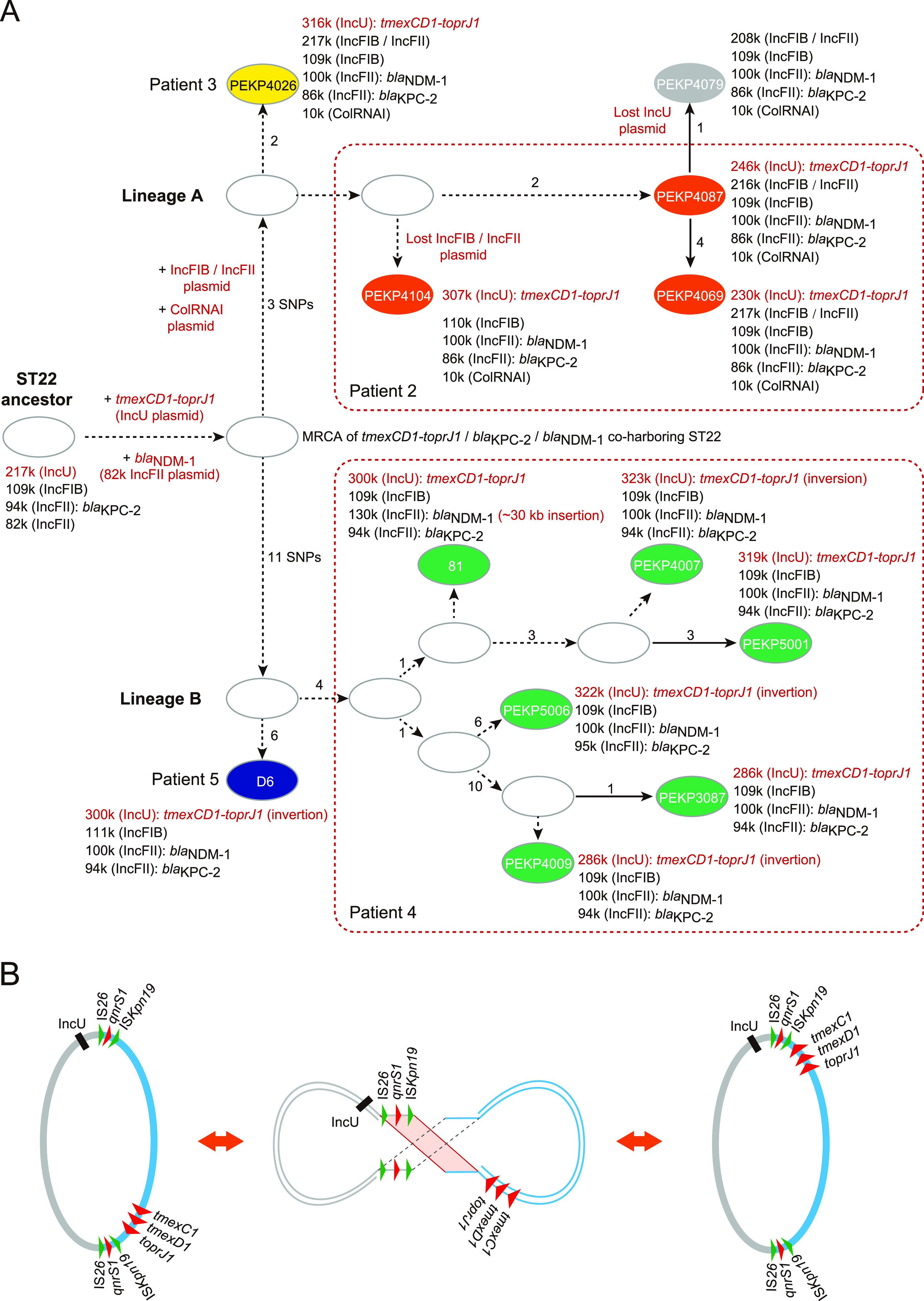
Inter- and intrahost microevolution of ST22 K. pneumoniae. (A) Evolutionary history of ST22 K. pneumoniae strains. The evolutionary sketch map was constructed by chromosomal SNPs first, and then the variations in plasmids and clinical information were used to illustrate the details. The number of SNPs and loss or acquisition of plasmids between two strains are marked. The plasmid composition is marked beside the strain names. The sequence variations of plasmids are also illustrated. (B) Sketch map exhibiting the occurrence of inversion caused by inverted repeats comprising IS*26*, *qnrS1*, and IS*Kpn19*.

We then reconstructed the evolutionary map by using chromosomal SNPs first and then used the variations in plasmids and clinical information to illustrate the details (Fig. 5). During inter- and intrahost evolution, plasmids acquire various AMR and/or heavy metal resistance genes (Fig. 4). In the ancestor ST22 isolate, whose genetic backbone was similar to PEKP3116 and PEKP3119, an insertion encoding *tmexCD1-toprJ1* might occur on the IncU plasmid, as well as the other insertion encoding *bla*_NDM-1_ on the IncFII plasmid, to become the most recent ancestor (MRCA) of the two ST22 lineages harboring *tmexCD1-oprJ1*. The two insertion events might occur together or gradually. This MRCA isolate might accumulate three SNPs to obtain a 10-kb ColRNAI-type plasmid and a 217-kb IncFIB/IncFII plasmid together or gradually to evolve lineage A, meanwhile accumulating 11 SNPs in parallel to evolve lineage B. An isolate of lineage A, PEKP4079, was transmitted from patient 2 to another patient, and the IncU plasmid seemed to be lost during interhost transmission.

Both lineages also exhibited intrahost population heterogeneity. Three lineage A isolates, PEKP4104, PEKP4087, and PEKP4069, were isolated from patient 2 for three consecutive months. There were 2 to 5 chromosomal SNPs among these phylogenetically close isolates, and the IncU plasmid bearing *tmexCD1-toprJ1* varied from ~230 to 307 kb in size, with large insertions, deletions, and rearrangements. PEKP4104 also lost the IncFIB/IncFII plasmid. Six lineage B isolates were isolated from patient 4 for three consecutive months. There were 1 to 20 chromosomal SNPs, and the *tmexCD1-toprJ1*-bearing IncU plasmid varied from ~286 to 323 kb in size. In addition, the long-fragment variations mainly occurred in two sites: one was related to the IS*26* element near the 3′ end of the sequence, and the other was related to the inverted repeats (IRs) comprising IS*26*, *qnrS1*, and IS*Kpn19*. There was only one copy of the sequence located near the IncU replicon in the ancestor plasmid, and its reverse complement was inserted downstream of *tmexCD1-toprJ1* in most of the *tmexCD1-toprJ1*-bearing IncU plasmids, except pPEKP4104-307 and pPEKP4069-230 of lineage A. The fragments between the two copies of IRs were ~182 kb in size and inverted in pPEKP4007-323, pPEKP5006-322, and pPEKP4009-286 of isolates from patient 4 and pD6-300 of isolate D6 from patient 5.

### Differential expression of *tmexCD1-toprJ1* contributed to tigecycline heteroresistance.

**FIG 6:**
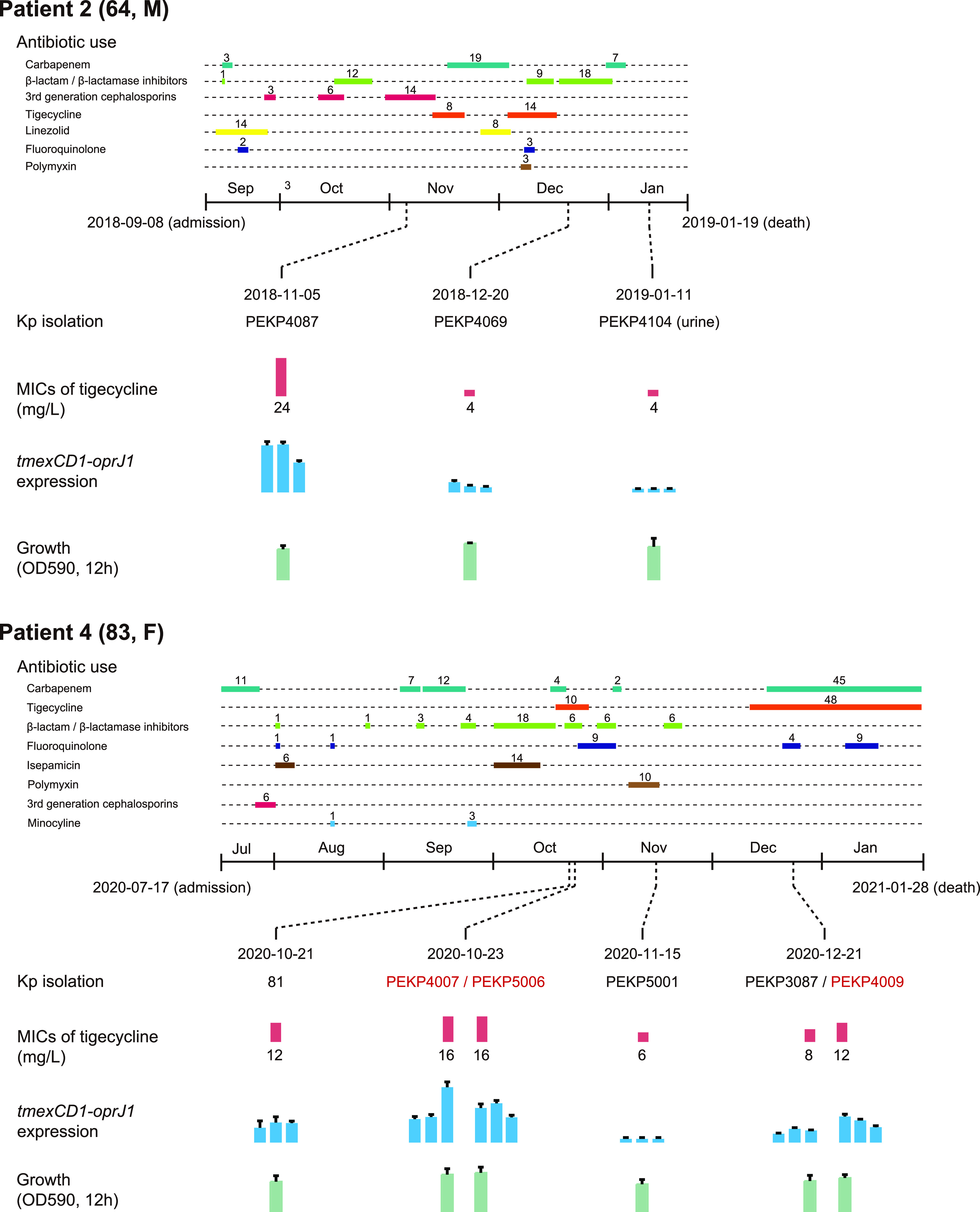
Heteroresistance of ST22 K. pneumoniae within patient 2 (A) and patient 4 (B). The use of antimicrobial agents, isolation, MIC, expression of *tmextCD1-toprJ1*, and OD_590_ values of culture for 12 h are displayed.

In line with the intrahost population heterogeneity, we also observed varied levels of tigecycline resistance among *tmexCD1-oprJ1*-harboring ST22 K. pneumoniae isolated from the same patients (Fig. 6; Table S2). The MICs of tigecycline varied from 6 to 16 mg/L against the isolates from patient 4 and 4 to 24 mg/L against the isolates from patient 2. The highest MICs were observed during treatment or pretreatment with tigecycline in patient 4 (16 mg/L against PEKP4007 and PEKP5006) and patient 2 (24 mg/L against PEKP4087). Interestingly, the MICs decreased against the isolates when tigecycline treatment was withdrawn for both patients and increased again in patient 4, who reused tigecycline (Fig. 6). By quantitative PCR (qPCR) detection of the *tmexCD1-toprJ1* genes, the copy numbers were approximately one per cell in all the isolates, indicating that no copy number variation occurred on the *tmexCD1-toprJ1*-bearing plasmid (DNA level). By reverse transcription-qPCR (RT-qPCR) detection of *tmexCD1-toprJ1* mRNA, we observed differential expression of these genes in the intrahost population (Fig. S3 and S4). In line with the variation in the MICs of tigecycline, the expression of *tmexCD1-toprJ1* exhibited the same trends. The growth of isolates was not significantly different within the two intrahost populations, indicating that there might not be significant fitness cost due to the presence and expression of *tmexCD1-toprJ1*. PEKP4087 exhibited the highest MIC (24 mg/L) of tigecycline and presented no significant differences in the growth curve compared with other isolates (PEKP4069, PEKP4104) isolated from patient 2. Moreover, isolate PEKP4007 from patient 4 presented higher MICs (16 mg/L) of tigecycline and a more prominent growth rate than the other isolates (Fig. S5). In addition, we observed an interesting phenomenon that the large fragment containing *tmexCD1-toprJ1* in the IncU plasmid might invert naturally. Among the assembled plasmid sequences, this region might be randomly inverted (Fig. 2). We designed a PCR experiment to detect the inversion in PEKP5001 and PEKP5006 and found that the inversion and original status both existed in the two isolates, although the assembled sequences were different (Fig. S6). We speculated that this region might be unstable and related to the differential expression of *tmexCD1-toprJ1* and different levels of tigecycline resistance.

## DISCUSSION

Here, from long-term surveillance within our hospital, we comprehensively demonstrated the clinical, epidemiological, and genomic characteristics of pandrug-resistant K. pneumoniae coharboring *tmexCD1-toprJ1*, *bla*_KPC-2_, and *bla*_NDM-1_. Importantly, the ST22 K. pneumoniae isolates caused infections in patients with poor prognosis. The full *tmexCD1-toprJ1* gene cluster was discovered in two types of plasmids harbored by K. pneumoniae strains of two different STs. Genetic recombination and acquisition occurred during inter- and intrahost evolution. Importantly, it is the expression of *tmexCD1-toprJ1* that plays a key role in the heteroresistance of tigecycline.

The *tmexCD1-toprJ1* and its subtype were mainly discovered among *Enterobacteriaceae*, including K. quasipneumoniae, Klebsiella variicola, Klebsiella michiganensis, and Proteus mirabilis (14, 15, 19). This efflux pump confers resistance to multiple drugs, including tigecycline, quinolones, cephalosporins, and aminoglycosides, in K. pneumoniae strains of animal origin and was then widely identified in K. pneumoniae strains derived from animals (13, 17, 18). To date, *tmexCD1-toprJ1*-harboring K. pneumoniae strains have also been discovered within various STs in hospitals, especially the endemic clones ST15 and ST11 (10, 14, 21, 22). Additionally, these genes were also detected in bacteria from various environmental samples, including urban drainage and food samples from market and slaughterhouse sewage (12, 16, 20). The spread of the *tmexCD1-toprJ1*-like gene cluster should be of great concern. Two *tmexCD1-toprJ1*-negative strains were isolated 3 months earlier than *tmexCD1-toprJ1*-positive strains in our hospital and belonged to a phylogenetically earlier lineage. In addition, it is reported that *tmexCD1-toprJ1* newly emerged into K. pneumoniae, but no *tmexCD1-toprJ1*-positive ST22 K. pneumoniae strains were reported previously. Therefore, we speculate that the *tmexCD1-toprJ1*-positive ST22 strains in our hospital evolved from the *tmexCD1-toprJ1*-negative strain by acquisition of the *tmexCD1-toprJ1*-harboring plasmid.

Of note, transposon 5393 (Tn*5393*) and IS*26* conferred *tmexCD1-toprJ1* acquisition. We also discovered the two types of genetic contexts of *tmexCD1-toprJ1*. In ST22 K. pneumoniae, the genetic element was flanked with Tn*5393* at the 5′ end, whereas IS*26* was flanked in ST3691 and ST11. Most previous studies confirmed that the plasmid harboring *tmexCD1-toprJ1* could be successfully transferred into E. coli (10, 14, 22). Importantly, it was confirmed that IS*26*, Tn*5393*, and ICE*KP* were conferred with *tmexCD1-toprJ1* acquisition and mobilization (9, 10, 16, 18). A previous study also demonstrated that the *tmexCD1-toprJ1* plasmid was unsuccessfully transferred into E. coli but successfully transferred into the hygromycin-resistant ST11 K. pneumoniae HS11286YZ6 (12). Conjunctive elements might play important roles in the rapid transmission of *tmexCD1-toprJ1* (10, 19, 22). In this study, we also discovered that the *tmexCD1-toprJ1* cluster was inserted into a nonconjugative IncU plasmid of ST22 K. pneumoniae via Tn*5393*, and ST3691 K. pneumoniae might have acquired *tmexCD1-toprJ1* bearing the IncFIB/IncHI1B plasmid via plasmid conjugation.

Most of the *tmexCD1-toprJ1*-like positive strains identified to date were recently discovered in China (9, 10), in addition to several reports of strains from Vietnam and Kenya (11, 16, 20). A previous study estimated that *tmexCD1-toprJ1* is rare in clinical K. pneumoniae strains in China (<0.1%) (9), and the positivity rate of animal-derived K. pneumoniae was 3.37% (12). Surprisingly, infection caused by ST3691 and ST11 was defined as community-acquired infection in our study. Furthermore, the plasmid harboring *tmexCD1-toprJ1* was stable after the 30th passage and tended to acquire heavy metal resistance genes during *in vivo* evolution, indicating that its persistent existence within the hospital environment might impel transmission and evolution, driving the emergence of the rapidly transferable “superbug.” Therefore, long-term genomic surveillance is essential, especially in areas with heavy antibiotic consumption.

Since the first report of *tmexCD1-toprJ1*, various MDR strains harboring *tmexCD1-toprJ1* and other AMR genes have been recovered (15). Importantly, the convergence and cotransmission risk of both resistance genes within endemic clones emerged (21). Sun et al. reported the coexistence of *tmexCD1*-*toprJ1* and *mcr-8* on the same plasmid in animal-derived K. pneumoniae strains (10). Moreover, it has been reported that CRKP strains coharbored the carbapenemase gene and *tmexCD1*-*toprJ1* in the same plasmid (20, 22). A previous study concluded that selective pressure imposed by the heavy use of older tetracyclines in China could have contributed to the emergence of this efflux pump (9). In this study, we found that ST22 K. pneumoniae became a PDR strain by acquiring and coharboring plasmids carrying *bla*_KPC-2_ and *bla*_NDM-1_ in the hospital and causing fatal infections.

A previous study reported that the K. pneumoniae strains presented with various MDR/virulence phenotypes during intrahost evolution, which might confer with different host response. During *in vivo* evolution, the pathogen population presented dynamic changes and intrahost heterogeneity under antibiotic and/or immune selective pressure (23). In this study, we found that the expression, but not the copy number, of *tmexCD1-toprJ1* contributed to the varied MICs of tigecycline when the population was under tigecycline pressure. We discovered genomic and phenotypical heterogeneity, whether within the same sample or among consecutive samples from the same host. Both the PEKP4007 and PEKP5006 strains isolated from the same sample (11 SNPs) showed increased MICs of tigecycline during *in vivo* evolution but presented a significant growth rate. PEKP4009 and PEKP3087 presented similar growth rates but displayed different MICs of tigecycline. A previous study reported that gene mutations were responsible for tigecycline or colistin resistance during treatment with tigecycline and polymyxin (24). However, we identified no mutation in *tmexCD1-toprJ1*. A previous study also demonstrated that gene expression changes triggered by ineffectual antibiotic treatment cause pathogens to transition between states of low and high virulence (25). An inversion in the IncU plasmid that caused *tmexCD1-toprJ1* to be relocated closer to the plasmid replicon might be responsible for the higher *tmexCD1-toprJ1* expression and higher level of tigecycline resistance. It has been confirmed that the genes that are closer to the origin site (*ori* proximal gene) are replicated first and are more highly expressed (26, 27). In E. coli, higher expression of oriC-proximal genes has been discovered on the chromosome, which is caused by the replication-induced increase of copy numbers (up to eight copies) of the oriC proximal region (28, 29). However, we did not find any evidence in previous studies about this phenomenon in the expression of genes in plasmids, which should be validated in the future.

In conclusion, by genomic surveillance, we discovered novel clinical *tmexCD1-toprJ1*-harboring K. pneumoniae subtypes and plasmids. Although the occurrence was rare, ST22 K. pneumoniae coharboring *tmexCD1-toprJ1*, *bla*_NDM-1_, and *bla*_KPC-2_ emerged in our hospital and caused fatal infection. Furthermore, in ST22 K. pneumoniae, the plasmid harboring *tmexCD1-toprJ1* displayed intrahost heterogeneity, and the inversion caused relocation of *tmexCD1-toprJ1* close to the replication origin of plasmid, which might be associated with the high expression of *tmexCD1-toprJ1*. These results call for further investigation of the prevalence of *tmexCD1-toprJ1* harboring K. pneumoniae, as well as intensive study of the mechanism of heteroresistance to improve antimicrobial treatment in the clinic.

## MATERIALS AND METHODS

### Patients and strains.

From 2017 to 2021, we conducted a cohort study at the Peking University Third Hospital to reveal the dynamic genomic epidemiology of Klebsiella spp. Clinical information of the infected patients was obtained from electronic medical records in the hospital. We calculated the Charlson comorbidity index (CCI) of the patients to evaluate the severity of illness and defined death or withholding life-sustainable therapy within 28 days as poor prognosis.

All the Klebsiella species isolates were stored in a −80°C freezer. The Klebsiella species isolates were initially confirmed by mass spectrometry and then by the Vitek 2 compact system. Finally, the Klebsiella species isolates were further identified by whole-genome sequencing (WGS) and analyses using Kleborate software (30). Isolates that harbored the *tmexCD1-toprJ1-like* gene cluster were analyzed together in the study. To clarify the unique genetic context of *tmexCD1-toprJ1*, we chose the same STs as the matched isolates, regardless of whether they were isolated from the same host.

### Antibiotic susceptibility test, hypermucoviscosity, growth curve, and biofilm formation capacity.

The antibiotic susceptibility test (AST) was performed using the Vitek 2 system as previously described according to the Clinical and Laboratory Standards Institute (CLSI) guidelines (2). The antimicrobial agents included ticarcillin-clavulanate (TCC), piperacillin-tazobactam (TZP), imipenem (IMP), meropenem (MEM), amikacin (AMK), tobramycin (TOB), aztreonam (ATM), cefepime (FEP), ceftazidime (CAZ), ciprofloxacin (CIP), levofloxacin (LEV), cefoperazone-sulbactam (CSL), trimethoprim-sulfamethoxazole (SXT), doxycycline (DOX), minocycline (MNO), tigecycline (TGC), and polymyxin (POL). The results were interpreted according to the CLSI guidelines (31), and the resistance breakpoints for tigecycline were interpreted by the EUCAST guidelines (version 1.1) (32). Hypermucoviscosity was determined by the string test (2). Isolates were grown at 37°C for 12 h and determined per hour by measuring the optical density at 590 nm (OD_590_). Each isolate was tested three times, and the growth curve was conducted by GraphPad Prism version 5. Biofilm formation capacity was evaluated in a 96-well plate by using crystal violet staining as previously described and interpreted by previous criteria (33).

### Plasmid transferability and stability.

To identify the transferability of plasmids carrying *tmexCD1-toprJ1*, we selected E. coli J53 as the recipient to conduct the conjugation experiment (12, 20). Briefly, the donor and recipient were mixed at a ratio of 1:1 and then cocultured in LB broth overnight. Subsequently, the mixture was spotted on MacConkey agar containing 1 mg/L tigecycline and 100 mg/L sodium azide. After overnight culture, the transconjugants were finally screened. To detect the plasmid stability, the *tmexCD1-toprJ1*-positive isolates were subcultured serially to the 30th passage (34). The descendants were also evaluated by AST and qPCR to determine the phenotype and genotype.

### Genomic DNA and RNA extraction and qPCR.

Whole-genome DNA was extracted using Tiangen magnetic universal genomic DNA kit (catalog no. DP705). To determine the copy number of the *tmexCD1-toprJ1* gene cluster, qPCR was conducted using NEB Luna universal qPCR master mix (catalog no. M3003S). The primers are listed in Table S4 in the supplemental material. To determine the expression level of *tmexCD1-toprJ1* in isolates continually isolated from the same patients, whole-genome RNA was extracted using Tiangen RNAprep pure cell/bacteria kit (catalog no. DP430), and RT-qPCR was also conducted using Qiagen QuantiTect SYBR green RT-PCR kit (catalog no. 204243). The PCR amplification consisted of the initial denaturation for 3 min at 95°C, followed by 35 cycles of denaturation for 30 s at 95°C, annealing for 30 s at 54°C, and extension for 30 s at 72°C, and then the final extension for 5 min at 72°C. We used the 16S rRNA gene as an internal control in PCR analysis.

### WGS and public data collection.

DNA previously extracted was further used for next-generation sequencing, and the libraries were prepared using Nextera technology as described in our previous study (2). Paired-end reads of 150 bp were generated by an Illumina NovaSeq 6000, and all the enrolled isolates were further sequenced by the Nanopore minION platform. Additionally, all of the ST22 and ST3691 genomes that were available in the GenBank database (28 genomes, accessed on 18 October 2021) were included in downstream analysis, resulting in 40 ST22 and 4 ST3691 genome sequences (Table S1). We also collected 18 previously published *tmexCD1-toprJ1*-positive plasmids (accessed on 18 October 2021) to understand their genetic contexts.

### Bioinformatic analysis.

Complete genomes were assembled by combining highly accurate short reads by Illumina sequencing and long reads by Nanopore sequencing via *de novo* assembly with a hybrid strategy according to published methods using Unicycler v0.4.4 (35). Primary gene prediction and annotation were performed by Prokka software as previously described (36). STs and serotypes were further detected by Kleborate software (30). Resistance genes, virulence genes, IS sequences, and plasmid replicon types were determined based on the comparison with ResFinder (37), Virulence Factor Database (38), IsFinder (39), and plasmidFinder (40) databases by BLAST.

To perform phylogenetic analysis, the complete chromosomal sequence of isolate PEKP3116 was used as the reference for analysis. The short reads of each isolate by Illumina sequencing were mapped to the reference by Bowtie 2 v2.2.8 first (41). The single nucleotide polymorphisms (SNPs) of each isolate compared with the reference were identified by using SAMtools v1.9, and the polymorphic sites among all isolates were combined together according to the reference genome using the previously published intrahost single-nucleotide variant (iSNV) calling pipeline (https://github.com/generality/iSNVcalling). We retained the high-quality SNPs (>5 reads of mapping quality >20) and removed the recombination sites detected by Gubbins v2.4.1 (42). Finally, the concatenated sequences of filtered polymorphic sites conserved in all genomes (core genome SNPs [cgSNPs]) were identified to construct the phylogenetic tree using the maximum-likelihood method by IQ-TREE v2.1.2 (43).

### Ethical approval statement.

All methods were performed in accordance with the Declaration of Helsinki. The protocol for this study was approved by the Peking University Third Hospital Medical Science Research Ethics Committee (M2021545). Due to the retrospective nature of the study, the need for approval was waived by the Peking University Third Hospital Medical Science Research Ethics Committee, and all the patient data enrolled in this study were anonymized.

### Data availability.

The genome sequences in this study were deposited into the NCBI database under BioProject accession no. PRJNA793277. The data sets used and/or analyzed during the current study are available from the corresponding author upon reasonable request.
