# Supplementary material for: Emergence and Inter- and Intrahost Evolution of Pandrug-Resistant Klebsiella pneumoniae Coharboring tmexCD1-toprJ1, blaNDM-1, and blaKPC-2
Source: Microbiol Spectr. 2023 Jan 31;11(2):e02786-22. doi: 10.1128/spectrum.02786-22 (PMC10100677; doi:10.1128/spectrum.02786-22)
Supplement: Supplemental file 1 — Supplemental material. Download spectrum.02786-22-s0001.pdf, PDF file, 2.5 MB [file spectrum.02786-22-s0001.pdf]

Supplementary Material

Emergence, Inter- and Intrahost Evolution of Pandrug-Resistant *Klebsiella pneumoniae Co*harboring *tmexCD1-toprJ1, bla*$_{NDM-1}$, and *bla*$_{KPC-2}$

Supplementary Methods

Enrolled Strains

From 2017 to 2021, we conducted a cohort study in the Peking University Third Hospital to unveil the dynamic genomic epidemiology of Klebsiella spp. in our hospital. Clinical information was obtained from electronic medical records, including basic demographic characteristics, underlying diseases, antimicrobial agent exposure, the site of infection, use of invasive devices, life-sustainable therapy (vasoactive drugs, mechanical ventilation and extracorporeal membrane oxygenation) and outcomes. Charlson comorbidity index (CCI) were calculated. Death or withhold life-sustainable therapy within 28 days was defined as poor prognosis.

Growth curve, biofilm, hypermucoviscosity and serum killing

Those *tmexCD1-toprJ1* positive plasmids in strains were unable to perform conjugation assays on, no transconjugants formed and used for the downstream phenotype experiment. Briefly, bacterial suspensions were collected and mixed with healthy human serum at the rate of 1:3. The mixture was agitated at 37°C and the number of the clone was recorded at time 0 and after 1h, 2h, and 3h of culture for 24 h, respectively[1]. Survival percentages at each different time were calculated and used to determine serum susceptibility/resistance followed by previous definition[2]. Hypermucoviscosity was determined by the string test[3]. Biofilm formation capacity was evaluated in 96 well plate by the crystal violet as previously described and interpreted by the previous criteria[4]. Strains were grown at 37°C for 12h and determined every per hour by measuring the OD590. The survival percentage and growth curve conducted by the GraphPad Prism version 5. Each strain was tested three times.

Detailed plasmid transferability and stability

To further understand the other AMR plasmid transferability within the *tmexCD1-toprJ1* positive strains, the *E. coli* J53 was also used as the recipient and the meropenem (8 mg/liter) was chosen as the screening antibiotics. The MICs of the transconjugants was detected and then sequenced by the Nanopore platform.

Detailed qPCR

The PCR amplification consisted of the initial denaturation for 3min at 95°C, followed by 35 cycles of denaturation for 30 s at 95°C, annealing for 30 s at 54°C, and extension for 30 s at 72°C, and then the final extension for 5min at 72°C.

Bioinformatic analysis

Raw Illumina sequencing data were de novo assembled using SPAdes v3.13 and annotated by the Prokka, as previously described[5]. STs， AMR genes, virulence subtype genes and its associates ICEKP, IS sequences and plasmid replicon types were initially determined by the Kleborate software[6] and further identified by ResFinder[7], Virulence Factor Database[8], IsFinder[9], and plasmidFinder[10] database.

Supplementary Results

Transmissibility and stability of the *tmexCD1-toprJ1* positive plasmids

The plasmid pPEKP4245-298 from ST3691 Kp successfully transmitted to *E. coli* J53 by the rate of 3.75×10^{-6}, and the transconjugant showed tigecycline resistance with the MIC of 4 mg/mL. The *tmexCD1-toprJ1* genes in transconjugant were confirmed by qPCR. However, no positive transconjugants were determined after three attempts for the ST22 Kp strains. Additionally, the 30th passage of the *tmexCD1-toprJ1* positive strains still presented with the tigecycline resistance phenotype and harbored the *tmexCD1-toprJ1* genes.

Co-harboring and variation of bla_{KPC-2} and bla_{NDM-1} bearing plasmids

Along with the *tmexCD1-toprJ1* bearing IncU plasmid, the tigecycline-resistance ST22 Kp also presented multiple IncF plasmids encoding bla_{KPC-2}, bla_{NDM-1} and heavy metal resistance genes (Figure 4). The *tmexCD1-toprJ1* negative ST22 carried four plasmids, the IncU plasmid without *tmexD1-toprJ1*, a 109kb IncFIB plasmid, an 82 kb IncFII plasmid without any resistance gene, and a 94 kb IncFII plasmid harboring bla_{KPC-2}. The IncFIB plasmids kept the size of 109~110 kb with minor changes in *tmexCD1-toprJ1* harboring ST22 Kp strains. In the strains acquired *tmexCD1-toprJ1* in IncU plasmid, the 82 kp IncFII plasmid obtained an insertion sequence encoding *sul1* and bla_{NDM-1} and the size increased to 100 kb as well (Figure 4A, 4B). The bla_{KPC-2} harboring IncFII plasmids varied in 86~95 kb (Figure 4C, 4D). In addition, four strains acquired another plasmid comprised the IncFIB and IncFII replicons (Figure 4E, 4F). This plasmid varied in 208~217 kb, and comprised the AMR genes including bla_{OXA-1}, *aac(6')Ib-cr* and *dfrA14*. This plasmid also encoded the genes conferring heavy metal resistances, including the *cus* and *cop/pco* operons conferring copper resistance, the *ars* operon conferring resistance to arsenate and arsenite, and *silE* gene conferring silver resistance.

Association of clinical *tmexCD1-toprJ1* harboring strains with the Kp in food chain

The *tmexCD1-toprJ1* harboring ST22 and ST3691 strains were both phylogenetically close to the strains of the same ST from our hospital. These results indicated that both *tmexCD1-toprJ1* harboring ST22 and ST3691 were origin from *tmexCD1-toprJ1* negative strains in our hospital by acquisition of *tmexCD1-toprJ1* bearing mobile genetic elements, however, the direct origin of these elements was not clear. We did not identify the ancestor IncU plasmid among published ST22 genomes. Interestingly, we also isolated one ST11 strain with truncated *tmexD1* and *toprJ1* (PEKP3038), and the plasmid was highly similar with some of the *tmexCD1-toprJ1* bearing plasmids widely distributed in Kp in food chain. ST11 Kp was widely studied and prevalent in hospitals, community and livestock industry. Moreover, the ST11 Kp harboring *tmexCD1-toprJ1* have been isolated from slaughterhouse in China[11]. We collected published ST11 genomes of Kp in China, and performed phylogenetic analysis together with ST11 strains in our hospital (Figure S3). The results showed that PEKP3038 were phylogenetically away from prevalent Kp clones in our hospital, and close to two ST11 Kp strains isolated from flies collected from a commercial chicken farm in Shandong province (Figure S3B), China from a previous study. Although these two strains were *tmexCD1-toprJ1* negative, *tmexCD1-toprJ1* harboring ST147 and ST256 Kp were identified from the strains isolated from chickens in the same poultry farm. The phylogenetic relationship and plasmid feature both revealed the ST11 Kp harboring truncated *tmexCD1-oprJ1* we obtained in hospital was highly related to those from food chain (Figure 3B).

Supplementary Reference

1. Liu C, Du P, Zhao J, et al. Phenotypic and Genomic Characterization of Virulence Heterogeneity in Multidrug-Resistant ST11 Klebsiella pneumoniae During Inter-Host Transmission and Evolution. Infect Drug Resist **2020**; 13: 1713-21.
2. Jousset AB, Bonnin RA, Rosinski-Chupin I, et al. A 4.5-Year Within-Patient Evolution of a Colistin-Resistant Klebsiella pneumoniae Carbapenemase-Producing K. pneumoniae Sequence Type 258. Clinical infectious diseases : an official publication of the Infectious Diseases Society of America **2018**; 67(9): 1388-94.
3. Liu C, Du P, Xiao N, Ji F, Russo TA, Guo J. Hypervirulent Klebsiella pneumoniae is emerging as an increasingly prevalent K. pneumoniae pathotype responsible for nosocomial and healthcare-associated infections in Beijing, China. Virulence **2020**; 11(1): 1215-24.
4. Liu C, Pan F, Guo J, et al. Hospital Acquired Pneumonia Due to Achromobacter spp. in a Geriatric Ward in China: Clinical Characteristic, Genome Variability, Biofilm Production, Antibiotic Resistance and Integron in Isolated Strains. Frontiers in microbiology **2016**; 7: 621.
5. Bankevich A, Nurk S, Antipov D, et al. SPAdes: a new genome assembly algorithm and its applications to single-cell sequencing. Journal of computational biology : a journal of computational molecular cell biology **2012**; 19(5): 455-77.
6. Lam MMC, Wick RR, Watts SC, Cerdeira LT, Wyres KL, Holt KE. A genomic surveillance framework and genotyping tool for Klebsiella pneumoniae and its related species complex. Nature communications **2021**; 12(1): 4188.
7. Bortolaia V, Kaas RS, Ruppe E, et al. ResFinder 4.0 for predictions of phenotypes from genotypes. The Journal of antimicrobial chemotherapy **2020**; 75(12): 3491-500.
8. Chen L, Yang J, Yu J, et al. VFDB: a reference database for bacterial virulence factors. Nucleic

acids research **2005**; 33(Database issue): D325-8.

9. Siguier P, Perochon J, Lestrade L, Mahillon J, Chandler M. ISfinder: the reference centre for bacterial insertion sequences. Nucleic acids research **2006**; 34(Database issue): D32-6.

10. Carattoli A, Hasman H. PlasmidFinder and In Silico pMLST: Identification and Typing of Plasmid Replicons in Whole-Genome Sequencing (WGS). Methods in molecular biology **2020**; 2075: 285-94.

11. Peng K, Wang Q, Yin Y, et al. Plasmids Shape the Current Prevalence of tmexCD1-toprJ1 among Klebsiella pneumoniae in Food Production Chains. mSystems **2021**; 6(5): e0070221.

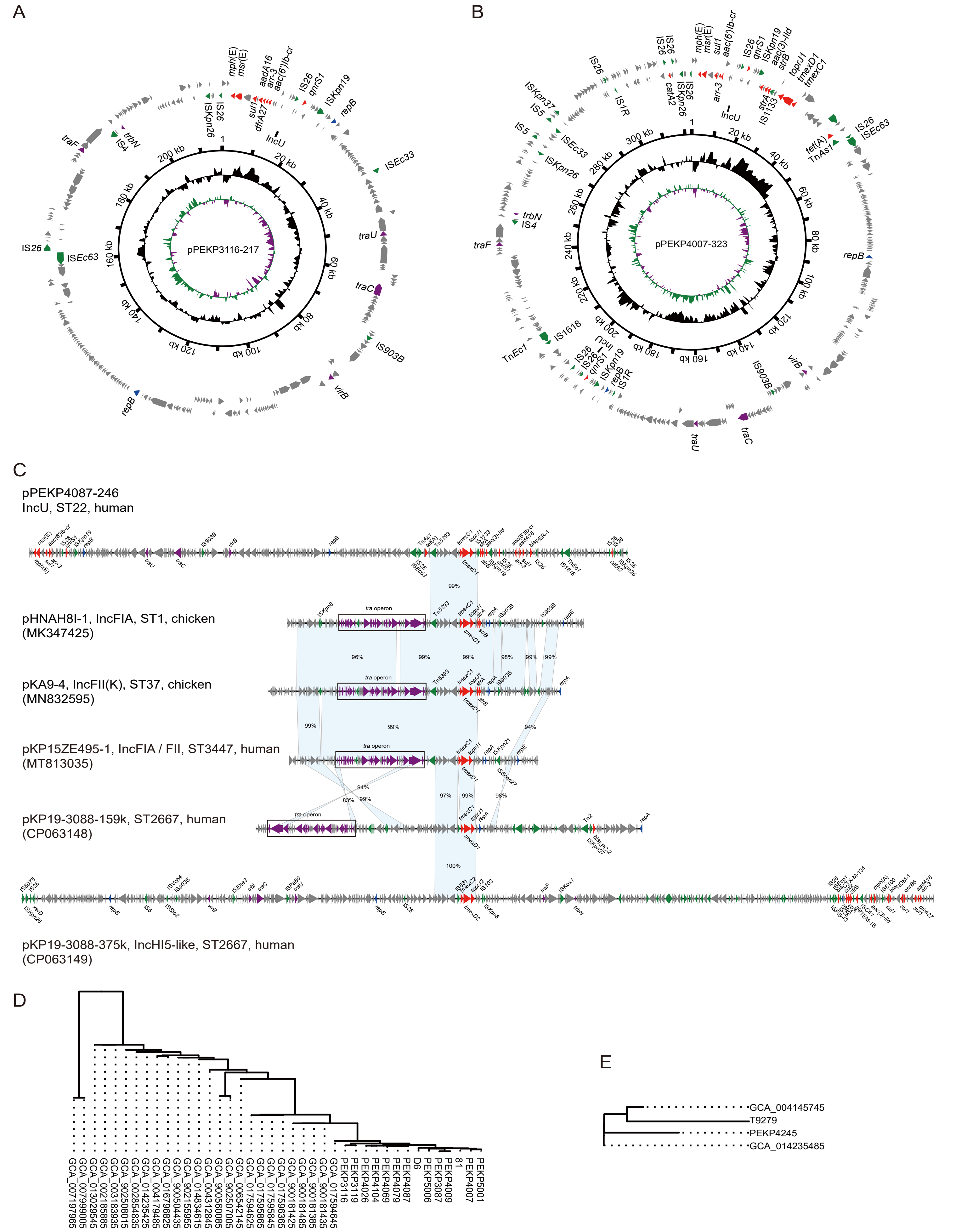

Figure S1. Molecular characteristics of IncU plasmids of ST22 Kp and alignments with published tmexCD1-toprJ1 bearing plasmids. A) and B) The circular sketch map and alignments of ancestor IncU plasmid and the one harboring tmex-CD1-toprJ1. C) Alignments with published tmexCD1-toprJ1 bearing plasmids. The matched regions between two sequences were displayed by light blue blocks and the identities were marked. The arrows represent the genes related resistance and transfer (red: AMR; green: integrase recombinase and transposase; purple: transfer associated; dark blue: plasmid replication; orange: heavy metal resistance; gray: other functions). D) Phylogenetic tree of our ST22 K. pneumoniae and published ST22 genomes. E) Phylogenetic tree of our ST3691 K. pneumoniae and published ST3691 genomes.

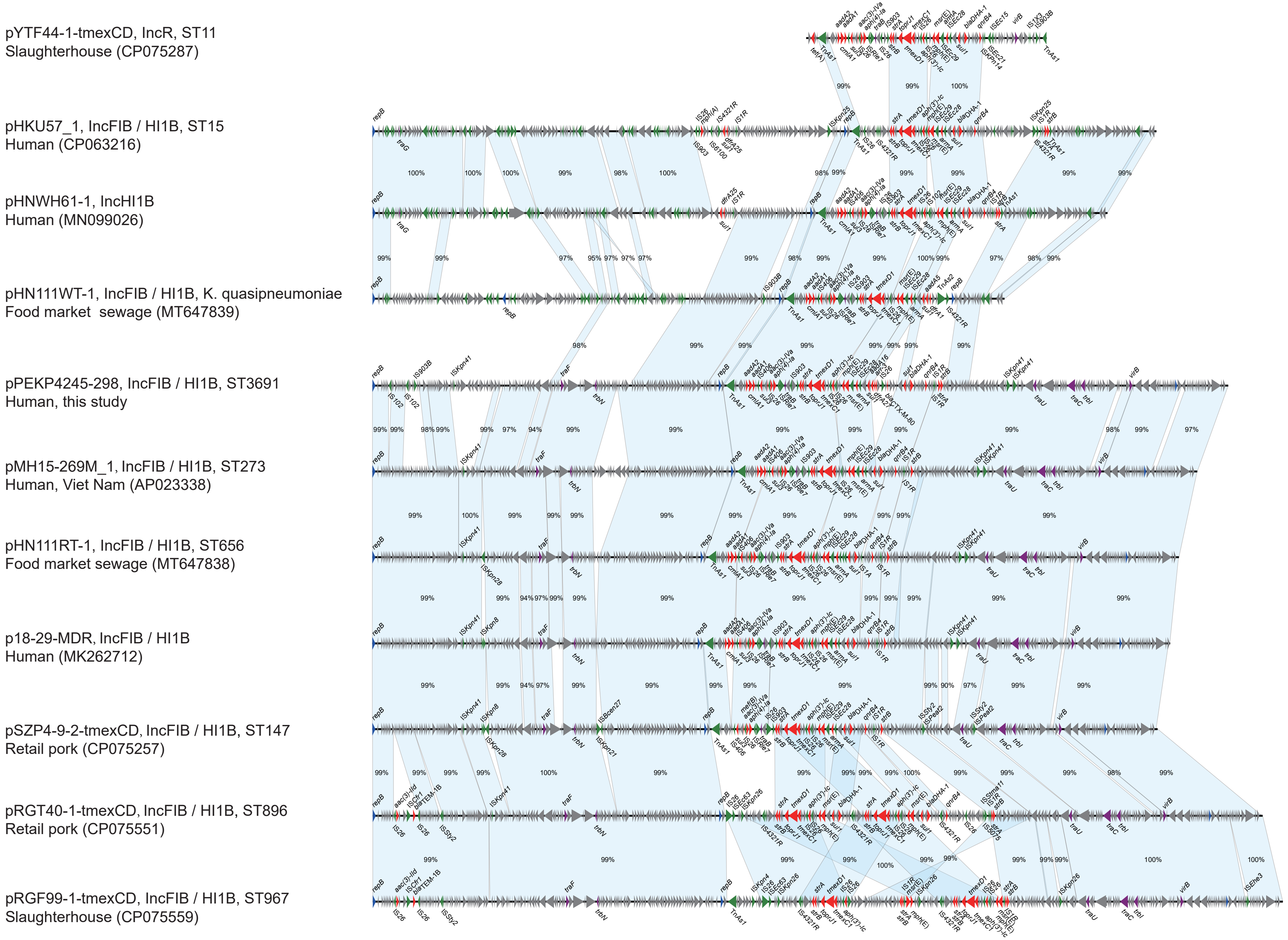

Figure S2. Alignments of tmexCD1-toprJ1 bearing from ST3691 Kp with published tmexCD1-toprJ1 bearing plasmids. The matched regions between two sequences were displayed by light blue blocks and the identities were marked. The arrows represent the genes related resistance and transfer (red: AMR; green: integrase recombinase and transposase; purple: transfer associated; dark blue: plasmid replication; orange: heavy metal resistance; gray: other functions).

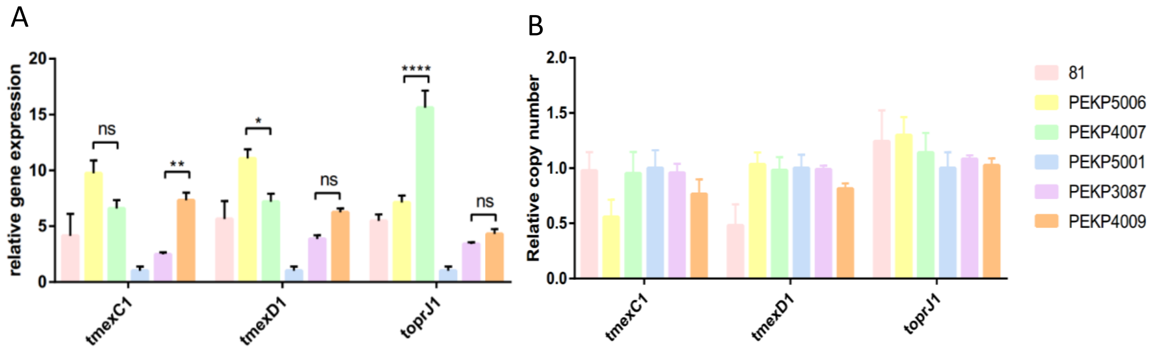

Figure S3. The expression and copy number of the tmexCD1-toprJ1 within the Patient 4 evolution. A) The RNA expression of the tmexCD1-toprJ1 within the Patient 4 evolution. B) The copy number of the tmexCD1-toprJ1 within the Patient 4 evolution.

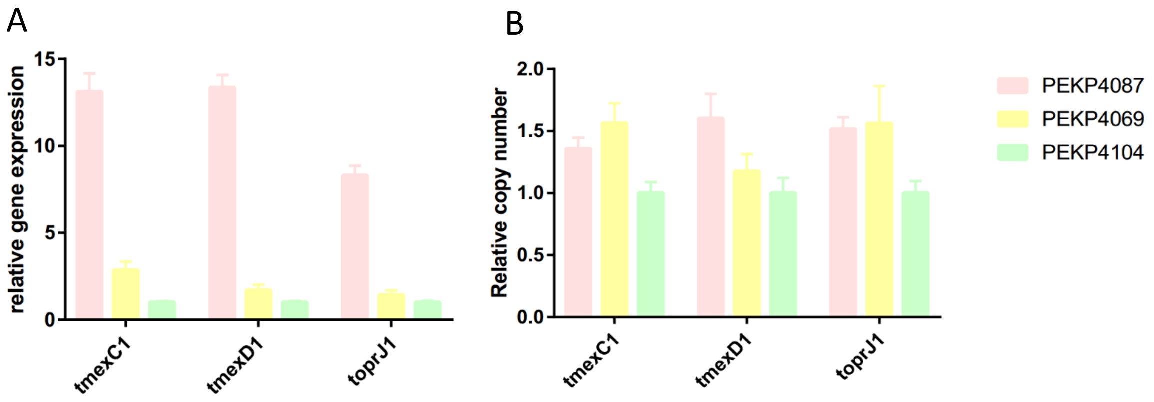

Figure S4. The expression and copy number of the tmexCD1-toprJ1 within the Patient 2 evolution. A) The RNA expression of the tmexCD1-toprJ1 within the Patient 2 evolution. B) The copy number of the tmexCD1-toprJ1 within the Patient 2 evolution.

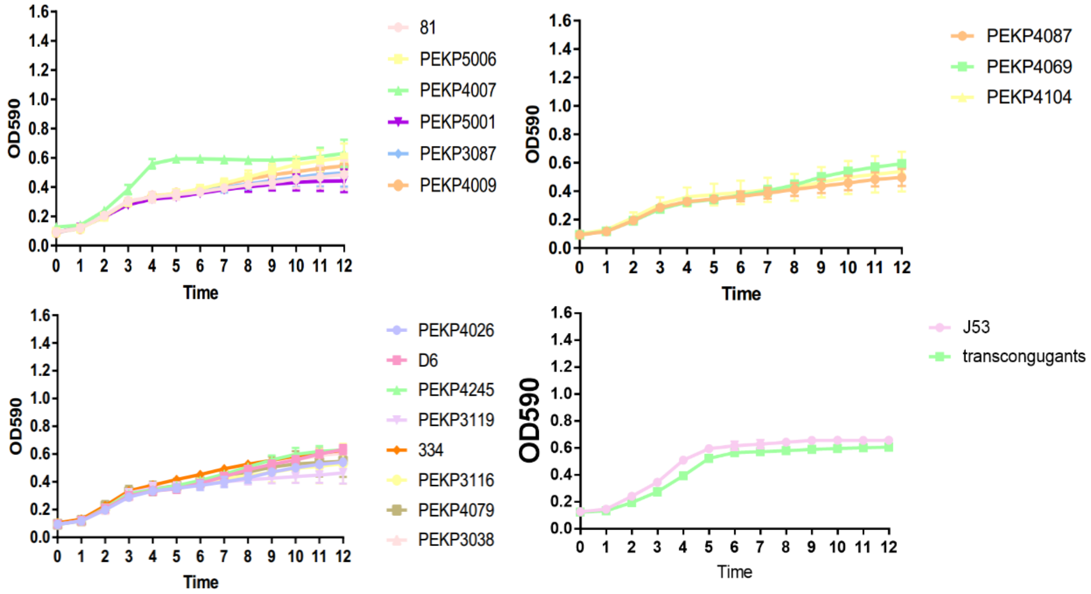

Figure S5. The growth curve of the enrolled Kp strains, E. coli J53 and transconjugant (tmexCD1-toprJ1 positive). A) The growth curve of the strains continuously isolated from the Patient 4. B) The growth curve of the strains continuously isolated from the Patient 2. C) The growth curve of other enrolled strains excepted the strains isolated from the Patient 2 and 4. D) The growth curve of the E. coli J53 and the transconjugant (tmexCD1-toprJ1 positive).

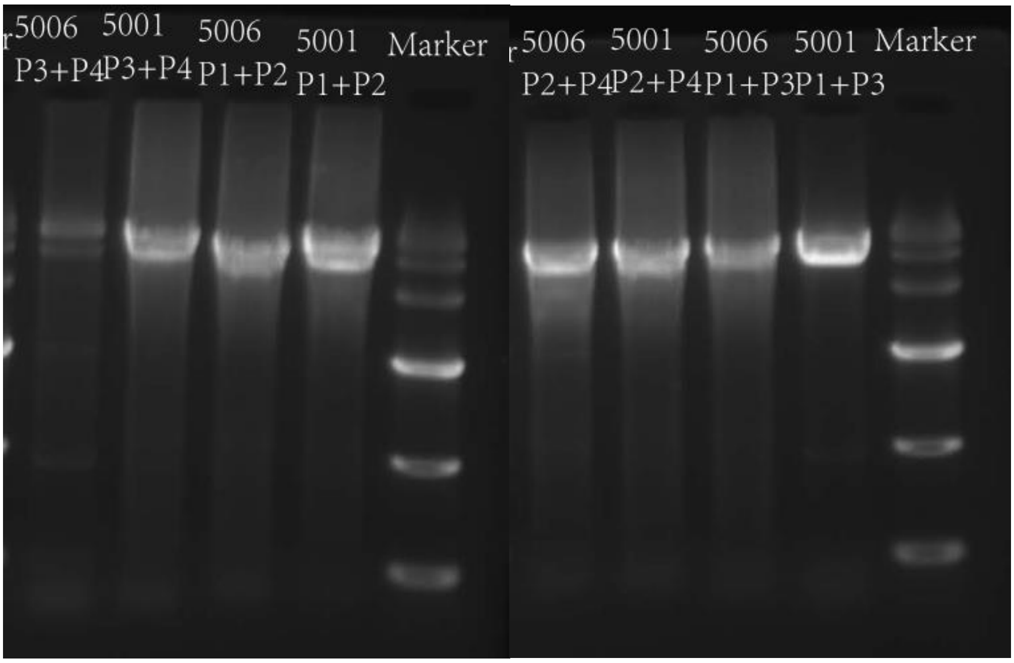

Figure S6. PCR detection of inversion.

Supplementary table 2. AST of the *tmexCD1-toprJ1*-like positive Kp strains and its transconjugant.

Items	PEKP4079	PEKP3038	**81**	**PEKP4007**	**PEKP5006**	**PEKP5001**	**PEKP3087**	**PEKP4009**	**PEKP4026**	**PEKP4087**	**PEKP4069**	**PEKP4104**	PEKP3116	PEKP3119	334	**D6**	**PEKP4245**	***E.coli* J53**	**Transconjugant^ (NDM+)**
CRO[#]	6	6	6	6	6	6	6	6	6	6	6	6	11	31	6	6	6	35	6
CZA[#]	13	22	11	14	14	15	14	6	6	15	15	13	8	30	29	13	15	32	12
TCC	≥128	≥128	≥128	≥128	≥128	≥128	≥128	≥128	≥128	≥128	≥128	≥128	≥128	≤8	64	≥128	≥128	≤8	≥128
TZP	≥128	64	≥128	≥128	≥128	≥128	≥128	≥128	≥128	≥128	≥128	≥128	≥128	≤4	8	≥128	32	≤4	≥128
CAZ	≥64	≥64	≥64	≥64	≥64	≥64	≥64	≥64	≥64	≥64	≥64	≥64	8	≤0.12	4	≥64	32	0.25	≥64
CSL	≥64	16	≥64	≥64	≥64	≥64	≥64	≥64	≥64	≥64	≥64	≥64	16	≤8	16	≥64	≥64	≤8	≥64
FEP	≥32	≥32	≥32	≥32	≥32	≥32	≥32	≥32	≥32	≥32	≥32	≥32	2	≤0.12	≥32	≥32	≥32	≤0.125	≥32
ATM	≥64	≥64	≥64	≥64	≥64	≥64	≥64	≥64	≥64	≥64	≥64	≥64	16	≤1	16	≥64	≥64	≤1	≤1
IPM	≥16	1	≥16	≥16	≥16	≥16	≥16	≥16	≥16	≥16	≥16	≥16	≥16	≤0.25	≤0.25	≥16	1	≤0.25	8
MEM	≥16	≤0.25	≥16	≥16	≥16	≥16	≥16	≥16	≥16	≥16	≥16	≥16	≥16	≤0.25	≤0.25	≥16	≤0.25	≤0.25	≥16
AMK	≥64	≤2	≥64	≥64	≥64	≥64	≥64	≥64	≥64	≥64	≥64	≥64	8	≤2	≤2	≥64	≥64	≤2	≥64
TOB	≥16	≥16	≥16	≥16	≥16	≥16	≥16	≥16	≥16	≥16	≥16	≥16	8	≤1	≤1	≥16	≥16	≤1	≥16
CIP	≤0.25	≥4	≥4	≥4	≥4	≥4	≥4	≥4	≥4	≥4	≥4	≥4	1	≥4	1	≥4	≥4	≤0.25	≤0.25
LVX	≤0.125	≥8	4	4	4	≥8	≥8	≥8	≥8	≥8	≥8	≥8	1	≥8	1	4	4	≤0.125	≤0.12

DOX	1	4	**≥16**	**≥16**	**≥16**	**≥16**	**≥16**	**≥16**	**≥16**	**≥16**	**≥16**	**≥16**	1	4	≥16	**≥16**	**≥16**	≤0.5	≤0.5
MNO	2	4	**≥16**	**≥16**	**≥16**	**≥16**	**≥16**	**≥16**	**≥16**	**≥16**	**≥16**	**≥16**	≤1	≤1	8	**≥16**	**≥16**	≤1	≤1
TGC*	1.5	0.75	**12**	**16**	**16**	**6**	**8**	**12**	**12**	**24**	**4**	**4**	2	0.5	1	**12**	**4**	≤0.5	≤0.5
COL	≤0.5	≤0.5	**≥16**	**≥16**	**≥16**	**≥16**	**≥16**	**≥16**	**≤0.5**	**≤0.5**	**≤0.5**	**≥16**	≤0.5	≤0.5	≤0.5	**≥16**	**≤0.5**	≤0.5	≤0.5
SXT	≥320	≥320	**≤20**	**≤20**	**≤20**	**80**	**40**	**40**	**≥320**	**≥320**	**≥320**	**≥320**	≥320	≥320	≤20	**≥320**	**≥320**	≤20	≤20

Blod font:tmexCD1-toprJ1 positive Kp strains; Gray background: tmexD1-toprJ1 positive Kp strains ;CZA: Ceftazidime/Avibactam; CRO: Ceftriaxone; CAZ: Ceftazidime; FEP: Cefepime; ATM: Aztreonam; IPM: Imipenem; MEM: Meropenem; TZP: Piperacillin/tazobactam; CSL: Cefoperazone/sulbactam AMK: Amikacin; TOB: Tobramycin; MNO: Minocycline; COL: Colistin; CIP: Ciprofloxacin; LVX: Levofloxacin; *TGC: The MIC of the Tigecycline was determined by the Etest; #:AST was evaluated by the K-B method; ^: Transconjugant presented with blaNDM positive and was further confirmed by the Nanopore sequencing.

Supplementary table 3. AST of 30th passages of the tmexCD1-toprJ1 positive Kp strains.

Items	PEKP4069	PEKP4087	PEKP4104	PEKP4009	PEKP3087	PEKP5006	81	PEKP3038	PEKP5001
CRO#	6	6	6	6	6	6	6	6	6
CZA#	15	22	23	15	13	6	13	26	6
TCC	≥128	≥128	≥128	≥128	≥128	≥128	≥128	≥128	≥128
TZP	≥128	≥128	≥128	≥128	≥128	≥128	≥128	32	≥128
CAZ	≥64	≥64	≥64	≥64	≥64	≥64	≥64	≥64	≥64
CSL	≥64	≥64	≥64	≥64	≥64	≥64	≥64	16	≥64
FEP	≥32	≥32	≥32	≥32	≥32	≥32	≥32	≥32	≥32
ATM	≥64	≥64	≥64	≥64	≥64	≥64	≥64	≥64	≤1
IPM	≥16	≥16	≥16	≥16	≥16	≥16	≥16	0.5	≥16
MEM	≥16	≥16	≥16	≥16	≥16	≥16	≥16	≤0.25	8
AMK	≥64	8	8	≥64	≥64	≥64	≥64	≤2	≥64
TOB	≥16	≥16	≥16	≥16	≥16	≥16	≥16	8	≥16
CIP	≥4	≥4	≥4	≥4	≥4	≥4	≥4	≥4	≥4
LVX	≥8	≥8	≥8	≥8	≥8	4	4	≥8	≥8
DOX	≥16	≥16	≥16	≥16	≥16	≥16	≥16	≥16	≥16

MNO	≥16	≥16	≥16	≥16	≥16	≥16	≥16	8	≥16
**TGC**	**≥8**	**≥8**	**≥8**	**≥8**	**≥8**	**≥8**	**≥8**	**2**	**≥8**
COL	≤0.5	2	4	≤0.5	≥16	≥16	≥16	≤0.5	≥16
SXT	≥320	≥320	≥320	≥320	40	≤20	≤20	≥320	40

Supplementary Table 4. Primers used in the study.

Items	Primers (5'-3')
16s rRNA	
Forward primer	TGGAGCATGTGGTTTAATTCGA
Reverse primer	TGCGGGACTTAACCCAACA
toprJ1	
1*-Forward primer	CTTCGATCAACGCCAATGCC
1*-Reverse primer	AGTTGCTGGTCACTTCCGAG
2*-Forward primer	ATCGCCTGCCTTCGATCAAC
2*-Reverse primer	CCGACCTGATAGTTGCTGGT
tmexD1	
1*-Forward primer	GACAAGAACGCCAACACCAC
1*-Reverse primer	AGACGGCGAATCTCGTTGTT
2*-Forward primer	CACGAAGCTCAACGACAAGC
2*-Reverse primer	GTAACCCTGGTCTTCCACCG
tmexC1	
1*-Forward primer	CCTGGGTTATGCCGAGGTTC
1*-Reverse primer	TACACCGGATCGAGTTGCTG
2*-Forward primer	CTGGGTTATGCCGAGGTTCG
2*-Reverse primer	GTACACCGGATCGAGTTGCT
inversion	
P1-Forward primer	TACCTGGCTATCACAGGCCGAGTTTAT
P2-Reverse primer	TGTTCTCACGCTTCTCTTTTTCCGATT
P3-Forward primer	TGGGCTTTGCTCAGTGCTACCTGTTCG
P4-Forward primer	TACGCTGCTGTCCCTAAAGCACCAATC

*: Two pair primers of all the target gene were designed.
